# Preparation of MED1(transcription mediator subunit) gene nanocarrier and its mechanism of action on liver cell regeneration in chronic acute liver failure

**DOI:** 10.1080/21655979.2021.1981756

**Published:** 2021-10-06

**Authors:** Jinwei Guo, Zhixiang Zhang, Jincan Zhu

**Affiliations:** Department of Infectious Diseases, Shenzhen Hospital, University of Chinese Academy of Sciences (Guangming), Shenzhen Guangdong Province, China

**Keywords:** Nanocarrier preparation, chronic acute liver failure, liver cell regeneration, mechanism of action

## Abstract

Liver failure has attracted attention in clinical work due to its high mortality, and the development of liver transplantation is restricted by various factors. Therefore, it is very important to carry out research on the mechanism of liver cell regeneration. This article has studied in depth the preparation of MED1 gene nanocarriers, collected human plasmids and cells through experimental materials and experimental instruments, and conducted comparative research on conventional culture. This question conducts a regeneration experiment on liver cells in chronic-onset acute liver failure, divides patients into an experimental group and a control group, and understands the recovery of liver function according to the screening of their plasma samples and separation of plasma. This article selects the commonly used clinical biological markers, such as Na+, AFP, Alb, CHE (serum cholinesterase) and other indicators to reflect the regeneration ability of liver function. The incidence of surgical complications in the control group, such as ascites, infection, bleeding, HE, hepatorenal syndrome, and hyponatremia were 71.3%, 87.4%, 16.1%, 41.4%, 19.5%, and 33.3%, respectively. Significantly higher than the experimental group, the difference was statistically significant (P < 0.05); while gender, age, PLT level and whether to use hormones, artificial liver or not there was no significant difference between the two groups (P > 0.05).

## Graphical Abstract

**Figure:**
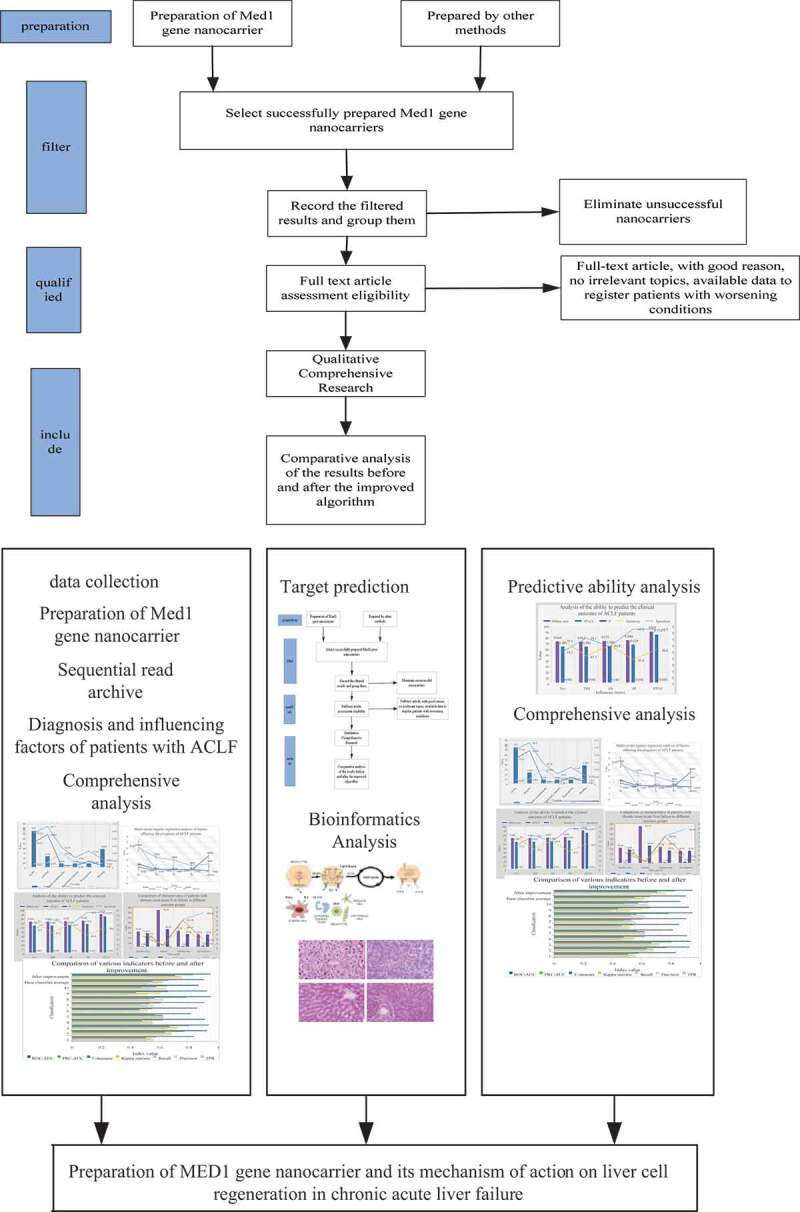

## 1. Introduction

Chronic acute liver failure (ACLF) is the most common severe end-stage type in CHB patients. ACLF has a dangerous condition, rapid progress, and poor prognosis. Although in recent years, great achievements have been made in the treatment of ACLF progress, but the fatality rate is still as high as 50%~60%. Mass degeneration and necrosis of liver cells caused by various reasons is its basic pathologically change. Liver transplantation is the main treatment method abroad. Due to the large number of patients in my country and the lack of liver donors, liver transplantation is difficult to popularize. Under this circumstance, whether hepatocytes can be regenerated in a timely and effective manner is the most important factor for the survival of ACLF patients. Therefore, exploring the mechanism of hepatocyte regeneration after ACLF is of great significance to the treatment of a large number of ACLF patients in my country.

‘Journal of Clinical Hepatobiliary Diseases’ pointed out that the liver has a strong ability to regenerate. Hepatocytes are terminally differentiated cells and are generally in a quiescent state. When a normal liver undergoes certain acute or chronic liver damage (such as liver tumors, partial liver resection, chemical or viral liver damage, etc.), the liver will immediately start the process of liver cell regeneration to achieve liver cell function compensation.

Since liver failure has become one of the important causes of human death, researchers have also launched research on it. Lodes U Acute hepatic failure (ALV) or acute and chronic liver failure (ACLV) is a multidisciplinary, high-risk disease. Objective to clarify the naming, pathophysiology, prediction, and environmental treatment options of ALV and ACLV, including the possibility of extracorporeal liver replacement therapy under the threshold of liver transplantation. Method narrative thinking, select literary research and representative cases. Results There are many causes of ALV and ACLV, and the fatality rate is high. ALV depends on the precise determination of the cause, because there are some special treatment facilities. Both ALV and ACLV may require liver transplantation to survive, and the indications and transplantation standards for ALV and ACLV are different. Although his research shows that liver failure requires transplantation, there is no clear explanation on how to perform transplantation and the difficulty of transplantation [1]. Ampuero J Acute-Chronic Liver Failure (ACLF) is a syndrome characterized by acute deterioration of liver function in chronic liver disease, accompanied by extrahepatic organ failure and high short-term mortality (15%). This concept breaks the traditional spectrum of liver diseases, especially liver cirrhosis. ACLF may appear in liver diseases ranging from compensatory to long-term cirrhosis. Despite the heterogeneity of the definition, it is clear that ACLF is caused by different types of precipitants in patients with chronic liver disease, and the prognosis is similar to that of acute liver failure. In this case, some new prognostic scores were proposed instead of MELD or Child-Pugh scores. A Portuguese retrospective study (N = 177) they conducted explored the usefulness of CLIF score to identify high mortality in patients with cirrhosis with ACLF5. But their research objects are not representative [2]. EH explored the effects of microribonucleic acid (miR)-34a on liver function and hepatocyte proliferation in the process of rat liver cell regeneration and its mechanism. They were randomly divided into sham 2 d group (2 days after hepatectomy) and sham 10 d group (10 days after liver resection), miR-34a-siRNA-2d group (miR-34a knockout + 2 days after liver resection) and miR-34a-siRNA-10d group (miR-34a knockout + 10 days after), each group 20 pieces. Serum alanine aminotransferase (ALT), aspartate aminotransferase (AST) and lactate dehydrogenase (LDH) levels were detected 2 and 10 days after operation. Take the rat liver to calculate the liver/body weight ratio. In addition, Feulgen staining method was used to detect the deoxyribonucleic acid (DNA) content of rat liver cells. Hematoxylin-eosin staining method was used to detect the pathological changes of rat liver. In addition, hepatocyte apoptosis in each group was detected by terminal deoxynucleotidyl transferase-mediated dUTP nick end labeling (TUNEL) staining. The expression levels of proliferating cell nuclear antigen (PCNA), Notch1 intracellular domain (NICD) and hypoxia-inducible factor-1 α (HIF-1 type α) Immunohistochemistry and Western blotting were used to detect miR-34a-siRNA in liver tissues of each group There were no significant differences in liver tissue between −2d group and Sham-2d group in ALT, AST, LDH, liver tissue pathological structure, hepatocyte apoptosis level, and expression of hepatocyte proliferating cell nuclear antigen. However, the expression levels of NICD and HIF-1 in the liver tissue of the α miR-34a-siRNA-2d group, compared with the sham 2d group, pMiR-34a knockout in the liver tissue can significantly improve liver function and liver cell regeneration. Its mechanism of action is to activate the Notch/HIF-1α signaling pathway. However, his research lacks the controllability of experimental research [3].

This paper mainly studies the preparation of med1 gene nano carrier and its mechanism of hepatocyte regeneration in chronic acute liver failure. Chronic acute liver failure often leads to serious damage or decompensation of liver synthesis, detoxification, excretion and biotransformation. The disease progresses rapidly, the clinical prognosis is very poor, and the short-term mortality is very high. The application of nano materials plays an important role in the treatment plan of promoting hepatocyte regeneration.The innovations of this paper are as follows: (1)MED1 gene nanocarrier carrier is applied to the study of hepatocyte regeneration for the first time, which provides a new idea and way for the clinical treatment of liver failure;(2) Theoretical research and empirical research. The combination of theory and research is verified by experiments.We believe that we can find a more accurate index and scoring system to reflect the prognosis of liver failure in the near future, which can dynamically evaluate the changes and prognosis of patients, and provide better treatment options for patients with liver failure.

## 2. Preparation of MED1 gene nanocarrier and its mechanism of action on liver cell regeneration in chronic acute liver failure

### 2.1 Chronic acute liver failure

Liver failure (Liver Failure) is the late manifestation of liver function. Due to various pathogenic factors, liver parenchymal cells and kupffer cells are severely damaged leading to systemic metabolic dysfunction and immune barrier disorders [4,5]. Liver failure can be divided into four categories: acute, subacute, chronic-acute, and chronic liver failure. Clinically, chronic-acute liver failure is the most common. HBV infection in my country is a common cause of chronic acute liver failure. ACLF has the characteristics of rapid disease progression, high mortality, and poor prognosis [6,7].

Chronic Acute Liver Failure (ACLF) is an acute liver damage syndrome, which occurs on the basis of chronic liver disease. In the process of liver synthesis, detoxification, excretion and biotransformation, it often causes serious decompensation obstacles.The short-term mortality rate is extremely high, and the application of nanomaterials to promote liver cell regeneration has a vital role [2,8]. Therefore, this article has conducted a large number of studies on the pathogenesis, diagnostic methods, treatment methods and prognostic factors of liver failure, but there is no unified liver cell regeneration model.

At present, some progress has been made in the treatment of chronic-onset acute liver failure at home and abroad, including comprehensive medical support treatment (antiviral therapy, liver protection, immune regulation, symptomatic support treatment, prevention and treatment of complications, etc.) based on individualized treatment of artificial liver Technology (plasma exchange, hemoperfusion, hemofiltration, hemodialysis, bilirubin adsorption and other non-biological artificial liver technologies used alone or in combination, biological artificial liver, mixed artificial liver), stem cell transplantation technology, G-CSF treatment Technology, artificial liver combined with stem cell transplantation technology, liver transplantation technology, etc [9,10]. Among them, foreign liver transplantation is the main treatment for liver failure; due to the large number of patients with chronic onset acute liver failure in my country, the relative lack of liver sources, expensive treatment costs, long-term immunosuppressive therapy after transplantation and other factors, liver transplantation Technical treatment of ALCF is difficult to be widely used in my country [11,12]. Under this circumstance, whether hepatocytes can be regenerated in a timely and effective manner is the most important determinant of survival of ACLF patients. Therefore, studying the regulation mechanism of liver cell regeneration after chronic acute liver failure is of great significance for the treatment of a large number of patients with chronic acute liver failure in my country [13,14].

### 2.2 Hepatocyte regeneration

Liver failure is the fastest growing, dangerous, and fatality rate among various liver diseases. Liver transplantation is still the most reliable treatment at present. Effective promotion of liver regeneration (Liver regeneration) may be the most effective medical treatment for liver failure in the future. In-depth study of the regeneration process of the liver, especially the exploration of key proteins related to liver regeneration, is important for the development of new promotion of liver regeneration and treatment of liver failure. The means are of great significance [15,16].

**Figure 1.**
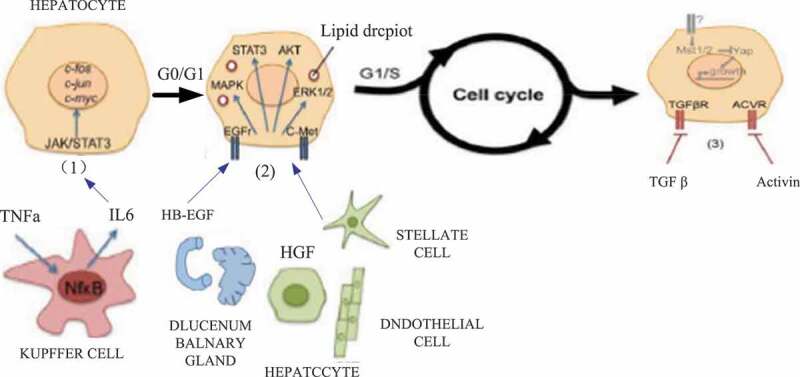
Hepatocyte regeneration process (Picture comes from Baidu Gallery)

The regeneration of the liver after injury includes the proliferation of mature hepatocytes and hepatic stem/progenitor cells, as well as the remodeling of the extracellular matrix (ECM). Previous studies on liver regeneration have mostly focused on the cell proliferation process, and very few studies on the remodeling process of ECM. ECM is mainly composed of structural proteins such as collagen, fibronectin, laminin, proteoglycan, and growth factors, inflammatory factors, etc., which are combined with them, and can provide the necessary microenvironment for cell adhesion, proliferation, differentiation and biochemical support. In addition, ECM is also involved in the formation of new blood vessels in regenerating organs [17,18]. In the early stage of the liver proliferative response after partial hepatectomy, there is a significant difference between the liver tissue morphology and the normal liver. The regenerated liver cells form cell clusters with 12–15 hepatocytes and avascular structure; while the content of ECM is less. It is significantly reduced due to a large amount of degradation and insufficient synthesis [19,20]. With the further development of liver regeneration, the proliferation of hepatocytes slows down, and the migration of stellate cells into the hepatocyte clusters leads to the formation of hepatic sinusoids and ultimately repairs the normal tissue structure of the liver. Studies have found that the remodeling of ECM is of great significance for liver regeneration. In the adult rat model of partial hepatectomy, injection of collagenase can significantly increase the proliferation of hepatocytes and the synthesis of liver DNA by HGF. The activation of MMP-2 and MMP-9 precursors after partial hepatectomy can promote the proliferation of hepatocytes by regulating the hepatocyte matrix environment. The differential regulation of ECM synthesis after partial hepatectomy can regulate the process of hepatocytes from the G0 phase to the proliferative phase and from the proliferative phase to the stationary phase [21,22]. Studies have found that TNF-α can induce the expression of MMP-9 to degrade ECM through the NF-kappa pathway, thereby regulating the G1/S phase transition and promoting the proliferation of hepatocytes. In summary, the remodeling of ECM is of great significance to the liver regeneration process [23–25]. The cysteine-rich acid secreted protein (SPARC) family in the extracellular matrix can regulate muscle differentiation, and SPARC is strongly expressed during tissue development and cell proliferation and differentiation. ECM is a member of the SPARC family.In-depth analysis of the dynamic changes of ECM-related genes in the process of liver regeneration will be of great significance for further identifying key proteins in the process of liver regeneration, clarifying the regeneration mechanism of the liver, and developing new research strategies to promote liver regeneration. The regeneration process of liver cells is shown in Figure 1.

### 2.3 MED1 gene

Transcriptional mediator subunit 1 (MED1) is one of the subunits of the transcriptional mediator complex (Mediator complex), and Mediator, as a main co-factor (masterco-factor), plays a bridge in the transcription process. The role, on the one hand, interacts with transcriptional factors (TFs) bound to enhancers (enhancer), and at the same time interacts with RNA polymerase II (RNA PolII) bound to promoters (promoter), thereby promoting downstream target genes Transcription.

MED1 plays a major role in the current clinical treatment. It can significantly inhibit cell growth. As a key co-activator of the estrogen receptor (ER), MED1 plays a key role in the ER-dependent gene expression and estrogen-dependent growth process, and participates in the resistance of another type of endocrine therapy drug Tamoxifen The formation process is highly correlated with the poor prognosis of patients. MED1 participates in the resistance formation process of classic endocrine drugs such as tamoxifen (Tamoxifen, TAM), and can be used in hormone-dependent breast cancer cells. It plays a role in the process of growth and proliferation, but the mechanism between the expression of MED1 and the regeneration of hepatocytes in patients with chronic acute liver failure has not been reported.

The transcription mediator complex participates in the process of gene replication and expression through a series of complex pathways. It consists of many subunits. Among them, MED1 is overexpressed in more than 50% of human breast cancer cases, and it is also known as HER-2. Amplicons, TRAP220, DRIP205, RB18, etc. can initiate gene transcription by binding to nuclear receptors. For example, after ER binds to estrogen, a specific region is exposed to bind to MED1 to form a pre-transcription initiation complex. In addition, MED1 can also initiate gene transcription by recruiting a variety of co-regulatory factors. It is one of the most studied subunits. MED1 and ER work together to participate in the process of cancer cell invasion and treatment resistance. Nanotechnology has shown good application prospects in the simultaneous use of chemotherapy and gene therapy, and has improved the problem of high cost. It has good stability, low solubility and insufficient selectivity during drug delivery. However, in order to effectively promote the clinical application of genes and nano-chemotherapy drugs, continuous efforts are needed.

### 2.4 Classification analysis concept

With the development of modern information technology, the information service technology of the medical industry must also keep up with the trend of the times, and ensuring the safety of patient information is also a top priority. First, we introduced association rules to express the relevance of patient data, and we are committed to improving the accuracy and applicability of association rules. Confidence:

(1) $$Con\left(X\to Y\right)=Q\left(Y\left|X\right.\right)=\frac{\left\{R:X\cup Y\subseteq R,R\in U\right\}}{\left\{R:X\subset R,R\in D\right\}}\times 100\%$$

It refers to the probability that Y appears at the same time under the conditions of X.

Support:

(2) $$Sup\left(X\to Y\right)=Q\left(X\cup Y\right)=\frac{\left|R:X\cup Y\subseteq R,R\in U\right|}{\left|U\right|}\times 100\%$$

This reflects the scope and importance of the relevant rules in all cases. The higher the support for association rules, the higher the adaptability and importance of the rules. The higher the trust, the more correct the rules. Generally speaking, the limits of support and trust are set by the user. Generally, the minimum trust threshold is used to filter the association rules acceptable to the user to achieve the minimum trust, where the minimum support limit is used to filter the association rules that the user accepts and reaches the minimum support.

Classification is based on the characteristics of the existing data set to establish a data mining technology used to map the data in the data set to the classification of a certain category. The classification mapping point is:

(3) $$C_{i}=Classify\left(Y_{i}\right)$$

The following four corresponding ratio formulas are introduced. In some past studies, one or several ratios will be used as the reference judgment standard:

(4) $$TNR=\frac{TM}{TM+TQ}$$

(5) $$FNR=\frac{FM}{FM+TQ}$$

(6) $$FPR=\frac{FQ}{TM+FQ}$$

(7) $$TPR=\frac{TQ}{FM+TQ}$$

It is used to evaluate whether a classification model prediction is accurate, the most widely recognized index in the industry is to comprehensively consider recall and accuracy. The ratio of the number of correctly classified positives to the actual total number of positives is called the recall rate.

(8) $$RE=\frac{TQ}{Q}$$

Also more intuitive is the concept of correct rate:

(9) $$ACC=\frac{TQ+TM}{C}$$

Error rate:

(10) $$ERR=\frac{FQ+FM}{C}$$

Improved classification algorithm Suppose T is a set of data samples, the capacity is set to u, n different classes V, the entropy required for the classification of a given sample is:

(11) $$Entropy(t_{1},t_{2},\cdots ,t_{n})=-\sum_{i=1}^{n}q_{i}\log_{2}\left(q_{i}\right)$$

Suppose the value set of non-categorical attribute B {a1, a2, …, av}, according to B, divide T into v subsets {S1, S2, …, Sv}, where Tj is the sample with value aj on B. tij is the number of samples of Ci in Tj, then the entropy of B divided by T is given by:

(12) $$Entropy\left(B\right)=-\sum_{j=1}^{v}\frac{t_{1j}+\cdots +t_{mj}}{t}Entropy\left(t_{ij},\cdots ,t_{mj}\right)$$

The information gain ratio is defined as:

(13) $$GainRatio\left(B,T\right)=\frac{Gain(B,T)}{SplitInf(B,T)}$$

Where Gain(B,T) is the information gain, and the definition is given by:

(14) $$Gain(B,T)=Entropy\left(t_{1},t_{2},\cdots ,t_{m}\right)-Entropy(B)$$

SplitInf(B,T) is the split information, which indicates the uniformity of the attribute split data, which is given by the following formula:

(15) $$SplitInf\left(B,T\right)=-\sum_{i=1}^{c}\frac{\left|T_{i}\right|}{\left|T\right|}\log_{2}\frac{\left|T_{i}\right|}{\left|T\right|}$$

## 3. Preparation of MED1 gene nanocarrier and its mechanism of action on liver cell regeneration in chronic acute liver failure

### 3.1 Preparation of Med1 Gene Nanocarrier

(1) Experimental materials

TRIReagentBD-American Sigma Company; Biopulverizer-American biospec; Mini-bead-Beater-16-American biospecRNasey MiniKit; Kit-German Qiagen; MiRCURYTM Array Power-Exiqon Company. Branched-chain polyethylene imine (bPEI, MW25KDa, anhydrous, produced by Aldrich-Sigma, USA); PEG derivative of clever functional group; Doxorubicin hydrochloride (Doxorubicin hydrochloride, D0X, HC1, Dalian Meilunsheng Technology Company production); C6-S-HyNic (C6-Succinimidyl □6-hydrazinonicotinate acetone hydrazone, □C6-SANH, produced by the U.S. company); bifunctional group PEG derivatives; membrane protein glands (Tryptone, produced by Beijing solarbio company)); Yeast extract (Yeast □extract, produced by Beijing solarbio company); Agarose (Spain BIO-WEST company); LipofectamineTM 2000 (produced by Rainbow Vitrogen company in the United States); Four seasons green fetal bovine serum (RBS, produced by Beijing solarbio company); RPMI1640 Medium (produced by Beijing Solarbio Company); DMEM medium (Gibico Company, USA); other reagents are analytical purity. □□

(2) Main instruments

MCO-17AC C02 incubator (Sanyo, Japan); particle size analyzer (Beckman Coulter, U.S.); flow cytometer (FACS caliber; dialysis bag (MWCO; 8000–14,000 Da, Beijing solarbio); constant temperature shaking culture Box (produced by Shanghai Zhichu struments company); FE20pH meter (Switzerland METTLE private company). Sampling gun-Gilson company of France; Micropipette tip-Axygen company of the United States; 15 ml centrifuge tube-Jiangsu Qilin Bell Instrument Manufacturing Co., Ltd.; 1.5 ml centrifuge tube-American Axygen company; 0.6 ml centrifuge tube-American Axygen company; 0.2 ml thin-walled eight-strip tube-American Axygen company.

(3) Plasmid

A eukaryotic plasmid (pDNA) donated by the Institute of Immunopharmacology and Immunotherapeutics of a certain university encodes enhanced green fluorescent protein (EGFP), and CMV is the driving promoter. The plasmid small extraction kit was used to extract pDNA expanded in DH5a Escherichia coli, and the operation was performed according to the kit instructions.

(5) Cell

Human lung adenocarcinoma cells (A549 cells) and human liver cancer cells (HepG2 cells) were provided by the Institute of Immunopharmacology and Immunotherapy of a certain university. Routinely culture A549 and HepG2 with a medium containing 10% FBS.

### 3.2 Chronic onset acute liver failure liver cell regeneration experiment

(1) Research object

The plasma samples collected in this article were collected from February 2019 to February 2020 in the Department of Infectious Diseases of a certain university. The initial diagnosis was chronic onset of acute liver failure. During treatment, patients whose condition deteriorated were diagnosed with delayed plasma sampling in internal or external wards. Before sampling, monitor patients with improved conditions in internal or external wards until liver function returns to normal. At the same time, comprehensive demographic and clinical data of patients are collected. Exclude patients with other viral infections (hepatitis A, C, D, E virus, HIV, EBV, cytomegalovirus), and exclude patients with drug-induced liver injury, autoimmune liver disease, and alcoholic liver disease. According to the guidelines of the ethics committee, the informed consent of the collected subjects was obtained.

Inclusion criteria:The initial diagnosis was chronic acute liver failure.

Exclusion criteria: exclude patients with other viral infections (hepatitis A, C, D, hepatitis E, HIV, EBV, cytomegalovirus), and exclude patients with drug-induced liver injury, autoimmune liver disease, and alcoholic liver disease .

(2) Experimental grouping

According to the clinical outcome, they are divided into experimental group and control group. The classification criteria are as follows: Experimental group: (1) Liver function returned to normal during hospitalization (2) Liver function regeneration improved but not completely returned to normal when discharged from the hospital, that is, liver cells are constantly growing, but the bad cells have not been completely replaced. Follow-up liver function after discharge until the liver function is normal; (3) The condition deteriorated when discharged from the hospital but He did not die, and the liver function returned to normal in the outpatient follow-up after discharge.The control group: (1) the condition deteriorated to late death during the hospitalization; (2) the condition deteriorated to the late stage at the time of discharge but did not die. The telephone follow-up after discharge confirmed the death and loss to follow-up; (3)) Liver function improved at discharge but not completely returned to normal. After discharge, outpatient or telephone follow-up confirmed that the condition deteriorated and was near death and death. Note: All patients who did not die were followed up for at least 24 weeks.

(3) Separate plasma from peripheral blood

Use EDTA anticoagulation tube to collect about 5 ml of fasting venous blood from the patient, leave it to stand for 1 hour at 4°C, centrifuge at 3000rpm for 10 min at room temperature, carefully draw the upper layer of plasma in a cryotube, and store it in liquid nitrogen for later use.

(4) Screening of plasma samples

We collected 64 patients who were first diagnosed with HBV-related chronic acute liver failure (ACLF) in the early stage, including 53 males and 11 females. The prognosis of 45 patients was confirmed by telephone and outpatient follow-up, and all patients who did not die were followed up for at least 24 weeks.; Experimental group: 36 cases, including 29 males and 7 females; Control group: 9 cases, all males; According to the median of each index in the two groups, we selected 3 patients in the experimental group and the control group for 2 hours A total of 12 plasma samples were spotted for miRNA gene chip detection.Use bioinformatics software to predict and select appropriate miRNA genes.

(5) Commonly used clinical biological markers

At present, prothrombin activity (PTA) and total bilirubin (TBil) are generally considered to be closely related to the prognosis of chronic acute liver failure. In addition, some studies believe that serum alpha-fetoprotein (AFP), cholinesterase (ChE), international normalized ratio (INR), serum sodium (NA+), albumin (Alb), blood ammonia, lactic acid, etc. are also related to the liver. Prognosis of failure is related.

It is generally believed that PTA is a poor prognostic factor for chronic acute liver failure. Patients with PTA between 10% and 20% have a higher mortality rate, while PTA<10% has a very low survival rate. A retrospective analysis showed that PTA is an independent risk factor affecting the prognosis of liver failure, and its cutoff value is 21%, which further indicates the predictive value of PTA<20% for poor prognosis of liver failure.

Low blood sodium is often seen in liver failure, dilution hyponatremia caused by water and sodium retention, and the use of loop diuretics are common causes of low blood sodium. Serum sodium is easy to obtain clinically, cheap and reproducible. In recent years, the influence of serum sodium on the prognosis of liver failure has received widespread attention. Serum Na+ is an independent predictor of death within 6 months of liver disease. Incorporating Na+ into the MELD model can provide a more accurate survival prediction than the MELD score, confirming the important effect of serum sodium on the prognosis of patients.

AFP, Alb, and ChE can all reflect the regenerative capacity of the liver. Some studies have shown that AFP is a prognostic and protective factor for liver failure, but AFP is closely related to tumors, and is mostly used as a positive marker of liver tumors, so it is for tumor patients or patients who cannot be ruled out., The application of AFP may be controversial.

ALT mainly exists in the cytoplasm of liver cells and is a sensitive sign of liver cell damage. It increases significantly in acute hepatitis, especially when bilirubin rises and transaminase decreases (choline separation phenomenon) ending.

The lactic acid level is not only related to the systemic inflammatory response syndrome, but also to the degree of multiple organ failure. The continuous increase in lactate level is a predictor of poor prognosis in fulminant liver failure, and the lactic acid combined with MELD score model is better than MELD score indicates that lactic acid has a good prognostic value in HBV-ALCF patients.

(6) Markers related to hepatocyte apoptosis and necrosis

Clinical pathology found that the basis of ACLF is that a large number of liver cell apoptosis and necrosis lead to a sharp decrease in the number of liver cells, which leads to a decline in liver synthesis and detoxification. The gold standard for judging the degree of liver disease is liver tissue biopsy, but it is an invasive procedure, and when liver failure occurs, the coagulation function is often abnormal, and the risk of bleeding is higher. Therefore, the current research is mostly noninvasive biomarkers, such as cytochromes, C (cytochromec, Cyt-c), microRNA (microRNA, miRNA).

Cyt-c is one of the most representative apoptotic signaling proteins in mitochondria, and it plays an important role in regulating cell energy metabolism. Hepatocyte necrosis and abnormal apoptosis are important links in the pathogenesis of acute liver failure.

### 3.3 Relationship between MED1 mRNA Expression and Patient Survival

Using the KaplanMeier-Plotter database, enter the search criteria: Cancer: Bresatcancer, Gene: MED1; Split patients by: median; Survival: DFS; Followupthreshold: All. Select different conditions, such as ER negative, ER positive, ER positive and receive endocrine therapy, and analyze the relationship between MED1mRNA expression levels and disease-free survival (DFS) in each group.

### 3.4 Statistical Analysis

Use SPSS24.0 software to analyze the data: use χ2 test, continuity correction, P value adopts two-tailed distribution, P < 0.05 means that the difference is statistically significant. Spearman rank correlation analysis is used to clarify the correlation between genes.

## 4. Preparation of MED1 gene nanocarrier and analysis of its mechanism of action on liver cell regeneration in chronic acute liver failure

### 4.1 Comparison of basic clinical data of the two groups of patients

The collected indicators that may affect the outcome of ACLF patients were included in the univariate analysis. The results are shown in Table 1. The levels of Alb, Na+, ChE, PTA, and ALT in the control group were 29.52 g/L and 135.91 mmol/L, respectively.3046.63 U/L, 32.45%, 598.64 U/L, all were significantly lower than the experimental group, the difference was statistically significant (P < 0.05); TBil, INR, SCr, and NH3 levels were 362.20, respectively 143.08 mmol/L, 2.53, 104.86 mmol/L, 53.52 mmol/L, all significantly higher than the experimental group, the difference was statistically significant (P < 0.05).

**Figure 2.**
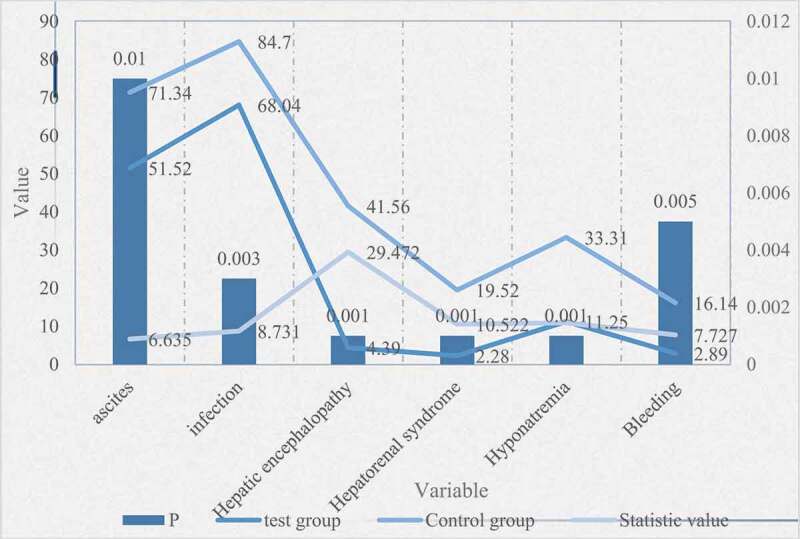
Comparison of complications between the two groups

**Table 1.** Comparison of basic clinical data between two groups of patients

variable	Test group	Control group	Statistic value	P
gender	55	74	3.34	0.066
age	48.52	48.81	0.12	0.912
hormone	10	7	1.42	0.236
Artificial liver	46	58	0.001	0.795
TBil	272	344	0.556	0.001
Alb	33	28	0.778	0.001
ChE	615.5	355	0.001	0.003
Na	3445.2	2955	2.313	0.022
SC	140.3	138	3.639	0.001
PLT	62.5	73	1.825	0.068
NH3	108.2	92	1.456	0.142
PTA	39.2	48	2.241	0.025
INR	38.36	34	2.928	0.003
GFR	1.94	2.26	2.55	0.012
Liver cirrhosis	45	59	1.614	0.114
ACLF type	-	-	0.493	0.481
A	27	26	4.541	0.156
B	10	5	2.986	0.003
C	35	54	4.512	0.001

As can be seen from Figure 2, the incidence of ascites, infection, bleeding, HE, hepatorenal syndrome, and hyponatremia were 71.3%, 87.4%, 16.1%, 41.4%, 19.5%, and 33.3%, respectively, and the control group was significantly higher than that In the experimental group, the difference was statistically significant (P < 0.05); while there was no significant difference in gender, age, PLT level, whether to use hormones, and artificial liver (P > 0.05).

**Figure 3.**
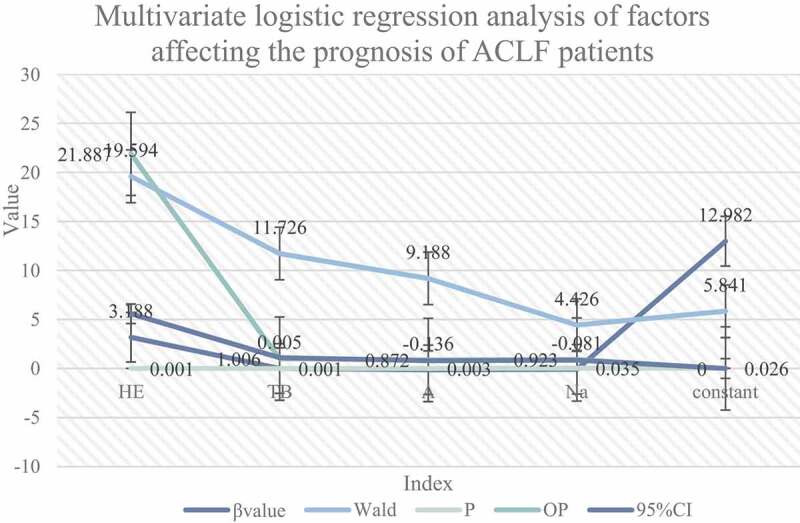
Multivariate logistic regression analysis of factors affecting the prognosis of ACLF patients

The indicators of P < 0.1 in Table 1 and Figure 2 were included in the multivariate logistic regression. The results are shown in Figure 3. The regression method was used to screen variables. The results showed that TBil, Alb, Na+, and HE were independent prognostic factors. Among them, hepatic encephalopathy, Total bilirubin is a risk factor (OR = 21.879, 1.007, P = 0.000, 0.001), and serum sodium and albumin are protective factors (OR = 0.906, 0.884, P = 0.021, 0.010).

**Figure 4.**
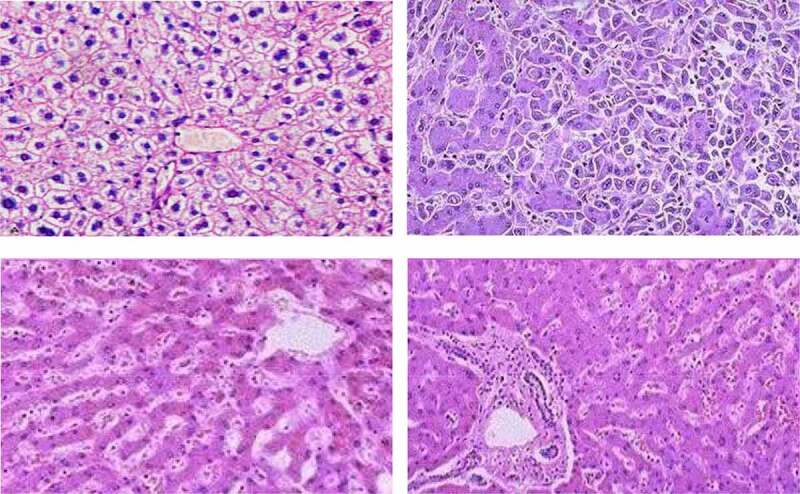
Liver cell regeneration pictures (Picture comes from Baidu Gallery)

Figure 4 shows the regeneration process of liver cells. In this study, it can be seen that the extracellular matrix is evenly distributed and neatly arranged in the cells; a large number of regenerated hepatocytes can be seen in PH24h, and a large number of regenerated liver cells can be seen in clusters, the nuclei of liver cells become shallow, and many mitotic figures are seen, and the collagen is significantly reduced, indicating that liver regeneration and proliferation are active, but the period ECM is largely degraded; compared with PH24h group, liver cell nuclear staining is still lighter in PH72h group, but the ECM content increases and liver cells are arranged more neatly, suggesting that with the further development of liver regeneration, ECM degradation decreases, synthesis speeds up, and participates in the structure of liver tissue. Remodeling; compared with the PH24h group, the hepatocyte nuclear staining in the PH7d group is deeper, indicating that the cell proliferation rate is slowed; collagen continues to accumulate in the liver tissue, and the collagen and hepatic cord arrangement are very close.

### 4.2 Diagnosis and influencing factors of ACLF patients

**Figure 5.**
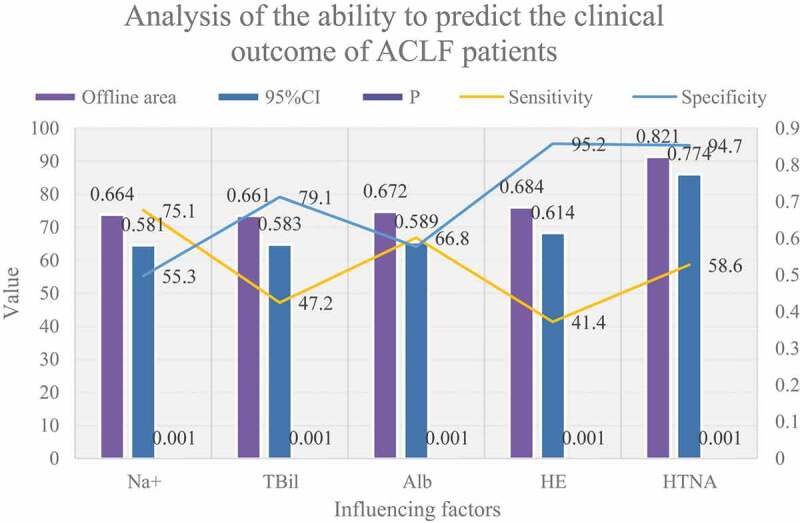
Analysis of the ability to predict the clinical outcome of ACLF patients

It can be seen from Figure 5 that ROC curve analysis shows that the best diagnostic points for TBil, Alb, and Na+ are 356.5umol/L, 30.5 g/L, and 137.5 mmol/L, respectively, indicating that when TB>356.5umol/L, Alb<30.5 g/ The prognosis may be poor when L and Na+<137.5 mmol/L. The AUC, sensitivity, and specificity of TBil, Alb, Na+, and HE in predicting ACLF prognosis were 0.664, 47.1%, 79.2%, 0.674, 66.7%, 64.6%, 0.667, 75%, 52.2%, 0.686, 41.4%, 95.8, respectively %. It can be seen that the area under the line and the specificity of the four joint predictions have been significantly improved, suggesting that the joint prediction can better predict the prognosis of patients with chronic acute liver failure.

Table 2 shows the test results of RNA samples. The quality inspection report shows that all RNA samples are Class A after inspection, and the quality meets the experimental requirements (RIN≥7), and subsequent experiments can be carried out.

**Table 2.** Sample Quality Inspection Report Form

Numbering	concentration	A260/280	A260/230	volume	Total	28S/18S	RIN	result
1	1.14	2.12	1.80	25	28.44	1.71	9.31	A
2	0.93	2.10	1.88	25	23.16	1.52	9.32	A
3	1.01	2.11	1.84	25	25.43	1.53	9.15	A
4	1.22	2.13	2.15	25	30.95	1.91	9.88	A
5	1.21	2.16	2.17	25	30.22	1.76	9.86	A
6	0.87	2.13	1.88	25	47.75	1.64	9.87	A
7	0.74	2.14	2.03	55	41.22	2.01	9.55	A
8	0.67	2.13	2.15	55	41.34	2.42	9.52	A
9	0.78	2.15	2.09	55	42.93	2.13	9.91	A

Table 3 shows the differential screening of liver regeneration mRNA. Before screening differential genes, probe filtering is performed. In each group of samples, at least one set of 100% probes labeled ‘P’ is left for subsequent analysis. For biological analysis, the difference between the effective P value obtained by the T test and the standard signal value is used. The standard is that the multiple differences are ≥2.0, and the P value is ≤0.05. In the non-biological iterative analysis, only the Foldchange value of multiple differences is used, and the standard is Foldchange value ≥ 2.0.

**Table 3.** Differential screening of liver regeneration mRNA

List number	test group	Control group	mRNA		
			Up	Down	Total
1	PH-24 h	SHAM	446	461	918
2	PH-72 h	PH-24 h	167	255	423
3	PH-72 h	SHAM	498	466	964
4	PH-7d	PH-24 h	765	261	1024
5	PH-7d	PH-72 h	325	63	375
6	PH-7d	SHAM	781	225	1088

### 4.3 mRNA expression level of MED1 is closely related to the expression level of HER-2 protein

We used the database to analyze the relationship between MED1 mRNA expression and clinical and pathological parameters in patients with chronic acute liver failure. The results are shown in Table 4: MED1 expression has nothing to do with the patient’s age, T stage, N stage, TNM stage, and ER/PR expression status. However, the high expression rate of MED1 in HER-2 protein-positive patients was significantly higher than that in the HER-2 protein-negative group (χ2 = 177.180, P = 0.000), and the difference was statistically significant. From the data in the table, it can be seen that MED1 mRNA expression The level is closely related to the expression level of HER-2 protein.

**Figure 6.**
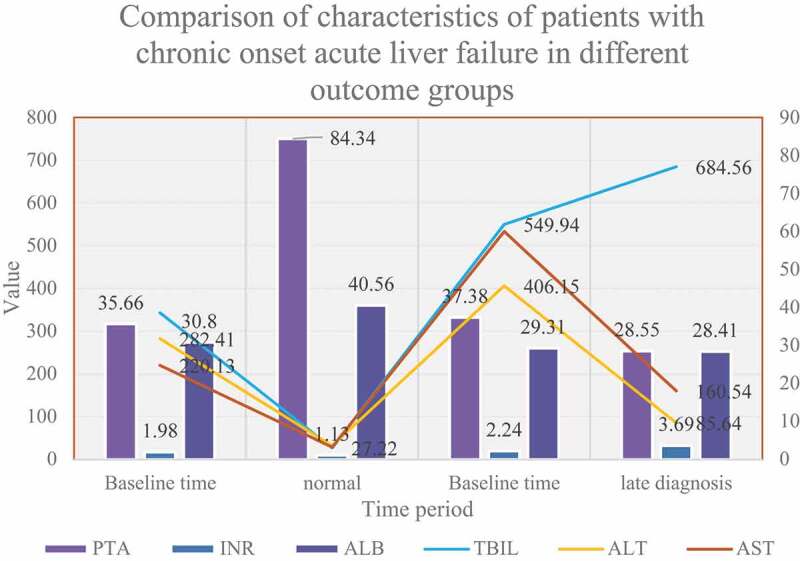
Comparison of characteristics of patients with chronic onset acute liver failure in different outcome groups

**Table 4.** Relationship between MED1 gene expression and pathological parameters of patients

clinical information	Number of cases	MED1 expression status		P
		Low expression	High expression	
Age <50	911	242	664	0.589
Age≥50	186	55	134	
T1+ T2	913	748	162	0.147
T3+ T4	174	155	25	
N0+ N1	875	731	148	0.294
N2+ N3	196	156	38	
TNM Phase I	182	156	24	0.251
Phase II	623	523	119	
Phase III	252	211	51	
Phase IV	23	18	3	
ER- and PR-	228	183	36	0.481
ER+ or PR+	138	119	28	
ER+ and PR+	692	574	115	
HER2 protein negative	596	554	38	0.001
Positive	162	82	76	

The specific clinical characteristics of the two groups of patients at different time points are shown in Figure 6. The two groups of patients used for miRNA gene chip detection had no significant differences in age (47.67–3.51VS48-4.58) and gender (all males).The two groups of ACLF patients had serum TBIL, PTA, and INR at baseline (initial diagnosis was early). There is no significant difference in the content of ALT, AST and ALB.

### 4.4 miRNAs and Their Target Genes Related to Liver Regeneration after ACLF

As shown in Table 5, we conducted bioinformatics analysis on candidate miRNAs and their target genes through the miRBase database. miRNAs such as hsa-miR-142-3p and hsa-miR-1297 may be related to liver regeneration after ACLF, and their biological functions Involved in the regulation of proliferation and apoptosis of various types of cells; at the same time, a total of 34 target genes such as EGFR, SMAD4, CXCR4, and PTEN may be related to liver regeneration after ACLF, and their biological functions involve participation in the proliferation and apoptosis of various cells regulation of death.

**Table 5.** Study on the biological functions of miRNAs and their target genes related to liver regeneration after ACLF

miRNA	Express the situation	miRNA function	Target gene	Target gene function
Hsa-miR-142-3p	The survival group was down-regulated compared with the death group at baseline	Inhibit the proliferation of various cancer cells and promote apoptosis	RAC1	Promote the proliferation of liver cancer cells
	The expression in the death group was down-regulated at baseline compared to when it was diagnosed as late		HGMGB1	Promote the proliferation of non-small cell lung cancer cancer cells and transplant their apoptosis
Hsa-miR-1297	The survival group was down-regulated compared with the death group at baseline	Inhibit the proliferation of liver cancer and human lung adenocarcinoma cells, and promote their apoptosis	TRIB2	Promote the proliferation of hematological malignant tumor cells and lung adenocarcinoma cells
			E2H2	
			HMGA2	

### 4.5 Comparison and analysis of results before and after the improved algorithm

**Figure 7.**
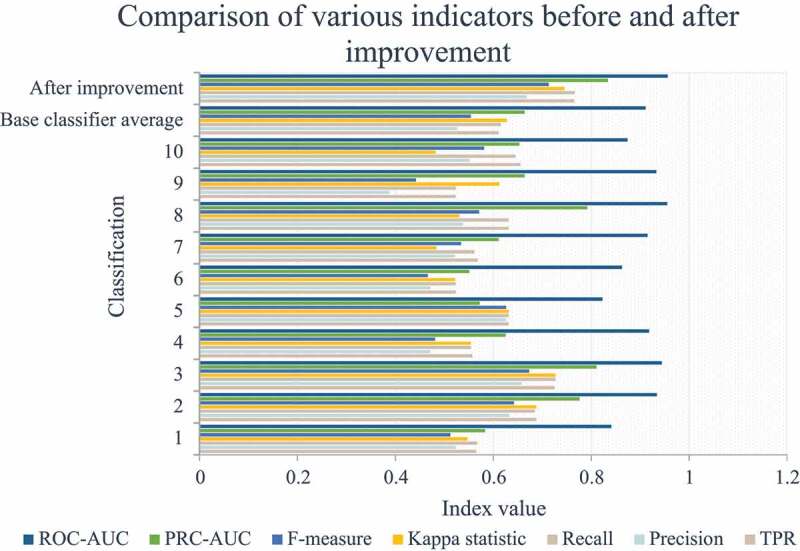
Comparison of various indicators before and after improvement

It can be seen from Figure 7 that the improved indicators have been significantly improved. Since the data set used for training is the data recognized by human experts, they are all positive samples. Therefore, when the positive and negative samples are distributed extremely unevenly, there is a problem of data label skew. Using F-Measure and Recall to judge classifier performance in practical engineering problems will be more meaningful than ROC-AUC. In addition, the PRC-AUC indicator is more meaningful than ROC-AUC in the case of unbalanced data tilt.

## 5. Insufficient research

The disadvantage of this article is that due to the individualization of patients, the causes and causes of liver failure, the course of disease, complications, and the different intervention and monitoring indicators used by different doctors, most of the clinical indicators are cross-sectional studies, and there is a lack of dynamic data. This greatly reduces the accuracy of the prognosis of liver failure. At present, large-scale clinical trials have not yet been carried out, which also makes them lack reliable basis and standard reference values, and more experimental data and clinical cases are needed to verify.

## 6. Conclusion

The subject of this article is the study of using MED1 gene nanocarriers for liver cell regeneration. Mainly study the preparation of MED1 gene nanocarrier and its mechanism of action on liver cell regeneration in chronic acute liver failure. Chronic acute liver failure often leads to severe damage or decompensation of liver synthesis, detoxification, excretion and biotransformation. The disease progresses rapidly, the clinical prognosis is extremely poor, and the short-term mortality rate is extremely high. The application of nanomaterials promotes liver cell regeneration. The treatment plan plays a vital role. The research in this article also confirmed the role of gene nanocarriers in promoting liver cell regeneration. The innovation of this paper is the first application of MED1 gene nanocarriers to the study of liver cell regeneration, which is an effective new treatment idea and method for anti-liver failure. The goal of this article is to enable more accurate indicators and scoring systems to dynamically assess the patient’s condition changes and prognosis, and to provide better treatment options for patients with liver failure.

## 7. Future research directions

With the advancement of science and technology and the spirit of seeking knowledge and exploration in medicine, we have a deeper understanding of the pathogenesis and disease progression of severe hepatitis and liver failure. We believe that we can find a prognosis that reflects liver failure in the near future. More accurate indicators and scoring systems can dynamically assess the patient’s condition changes and prognosis, and provide better treatment options for patients with liver failure.
